# Opioid-specific risk of respiratory depression in non-cancer pain: a retrospective cohort study

**DOI:** 10.1186/s12916-026-04972-z

**Published:** 2026-07-08

**Authors:** Carlos Raul Ramirez Medina, Mark Lunt, William G. Dixon, Meghna Jani

**Affiliations:** 1 Centre for Epidemiology Versus Arthritis, Centre for Musculoskeletal Research, The University of Manchester, Manchester, UK; 2 Division of Informatics, Imaging and Data Science, The University of Manchester, Manchester, UK; 3 NIHR Manchester Biomedical Research Centre, Manchester University NHS Foundation Trust, Manchester Academic Health Science Centre, Manchester, UK; 4 Department of Rheumatology, Salford Royal Hospital, Northern Care Alliance, Salford, UK

**Keywords:** Pain management, Drug-related side effects and adverse events, Pharmacoepidemiology, Respiratory depression, Opioids, Opiates

## Abstract

**Background:**

Opioids are associated with serious adverse outcomes, including premature death. Respiratory depression is among the most severe opioid-related events, yet data on its incidence in non-cancer pain remain limited. Pharmacological differences suggest varying respiratory risks across opioid drugs. This study evaluated the comparative risk of respiratory depression by opioid drug and dose, and the impact of concomitant gabapentinoids and benzodiazepines in hospitalised patients with non-cancer pain.

**Methods:**

A retrospective cohort study was conducted using electronic health records from a large tertiary hospital in Northwest England. Adult inpatients (≥ 18 years) receiving opioids for non-cancer pain were included. Opioid exposure was defined from drug administration records. Respiratory depression was identified using National Early Warning Scores or naloxone administration. Incidence rates were estimated by time-varying opioid exposure, opioid drug, and daily morphine milligram equivalent (MME). Associations with incident respiratory depression were examined using a Cox regression adjusted for confounders. Effect modifiers including co-administration of gabapentinoids or benzodiazepines were assessed for their impact on respiratory depression risk, in addition to opioids.

**Results:**

Among 32,909 inpatients, fentanyl (HR: 3.36, 95% CI 2.70–4.18), combination opioids (HR: 2.74, 95% CI 2.38–3.15), oxycodone (HR: 2.10, 95% CI 1.74–2.54), and morphine (HR: 1.84, 95% CI 1.59–2.12) were associated with a significantly higher risk compared with codeine. Relative to morphine, fentanyl (HR: 1.85, 95% CI 1.50–2.27) and combination opioids (HR: 1.49, 95% CI 1.32–1.69) remained associated with higher risk. Concomitant opioid-gabapentinoid use was associated with an increased risk compared to opioid use alone (HR: 1.73; 95% CI: 1.53–1.96). Doses ≥ 120 MME/day doubled the risk compared with < 50 MME/day (HR: 2.06, 95% CI 1.81–2.35), with evidence of increased risk at lower doses (31–60 MME/day) when examined using narrower dose categories.

**Conclusions:**

Our study highlights the increased risk of respiratory depression associated with specific opioid drugs, particularly fentanyl and combination opioids, compared with codeine or morphine. Even moderate doses (31 to 60 MME/day) were associated with an increased risk, while co-prescription of gabapentinoids with opioids was associated with a higher risk compared with opioids alone.

**Supplementary Information:**

The online version contains supplementary material available at 10.1186/s12916-026-04972-z.

## Background

Over the past few decades, the use of prescribed opioids for the treatment of non-cancer pain has increased considerably across North America [1] and Europe [2–7]. In response to growing safety concerns and overdose deaths, the U.S. Food and Drug Administration has issued several boxed warnings to highlight the serious risks associated with opioid use [8]. Fatal overdoses, up to 80% of which are unintentional [6], are most often caused by the respiratory depressant effects of opioids [9], leading to hypoxia, hypercapnia, and potentially progressing to cardiorespiratory arrest and death.

Although all potent opioid analgesics act primarily via the µ-opioid receptor system, they differ in their effects on respiratory control [10]. Opioids have distinct pharmacological actions depending on drug type, which may have differential effects on respiratory depression. For instance, tramadol is a weak µ-opioid receptor agonist with a proportion of its effect mediated by acting as a serotonin and norepinephrine reuptake inhibitor. Buprenorphine is a partial µ-opioid receptor agonist and a weak κ-opioid receptor antagonist. Whilst theoretically both tramadol and buprenorphine may hypothetically lead to less opioid-induced respiratory depression, severe cases have been reported for both drugs [11]. Furthermore, frequently prescribed concomitant drugs such as benzodiazepines and gabapentinoids are known to be associated with a substantially higher risk of opioid-related death [12, 13], This consequence is likely to be mediated through their known effects on drug-induced respiratory depression, but is infrequently examined.

Evaluating respiratory depression in opioid-treated patients presents several challenges. First, definitions vary widely across studies, from strict criteria based solely on administration of overdose reversal medicines such as naloxone, to broader assessments using case note reviews, post-operative capnography, or administrative codes for opioid poisoning or altered consciousness [14–16]. As a result, reported incidence rates range from 0.04% to 46%, depending on the proxies used (such as naloxone administration, hypoxaemia, bradypnoea, or other respiratory adverse events), and the populations studied [14, 15, 17–22]. Second, many studies use insurance claims or prescription records as a proxy for opioid exposure, assuming that intention to treat equates to actual drug use. However, because opioids are often prescribed on an ‘as required’ basis for pain management [23], this approach may lead to exposure misclassification and risk misattribution if drugs were prescribed or dispensed but not actually administered. Third, most previous research has focused on evaluating opioid-induced respiratory depression exclusively in post-operative patients [14, 15, 17–19, 24], thereby limiting the generalisability of findings to other inpatient populations.

Respiratory rate is routinely measured across inpatient settings as part of standard clinical monitoring. Early warning systems, such as the National Early Warning Score (NEWS) introduced by the Royal College of Physicians in 2012, standardise the recording, scoring and response to changes in physiological parameters. Increasingly captured digitally, these observations are valuable for population health research. Using such routinely collected clinical observations as an indicator of respiratory depression in hospitalised patients can reduce outcome misclassification compared with proxies such as retrospective coding (e.g., ICD-codes). Moreover, inpatient data allow for a more accurate definition of opioid exposure, as it includes drug administration records (i.e., whether a drug was actually given to the patient by a nurse, rather than prescribing information alone) and include detailed dosage information using electronic (e)-prescribing systems. This is especially important for medications such as opioids, where there is often variability in the prescription instructions in terms of the quantity of tablets that can be administered [23]. 

The aims of this study were to (i) evaluate the incidence of respiratory depression in opioid users for non-cancer pain; (ii) assess the comparative risk of respiratory depression across different opioid types and the comparative risk of daily dose; (iii) assess the effect of concomitant gabapentinoids and benzodiazepines on the outcome.

## Methods

### Study design and setting

We conducted a retrospective cohort study using secondary care electronic health records (EHRs) from a large tertiary care hospital in the Northwest of England. The study period spanned from September 26, 2014, to October 24, 2017. All medications were prescribed electronically with information on whether each drug was administered to the patient. Since September 2014, vital signs are recorded electronically every four hours in all hospital inpatients.

### Study population

We identified adult inpatients aged ≥18 years who were opioid users during their hospital admission. To define a cohort based on opioid use for non-cancer indications, we excluded patients with a current or prior malignancy diagnosis using ICD-10 codes, as cancer patients are often prescribed opioids for palliative or cancer-related pain, which may involve different prescribing patterns and distinct clinical risk profiles for respiratory depression compared to non-cancer patients.

Patients who were administered methadone or sublingual buprenorphine were excluded due to the frequent use of these medications in opioid substitution therapy for substance use disorders. We also excluded those who received intravenous opioids used exclusively for anaesthetic purposes (e.g., remifentanil), as these are typically administered in closely monitored surgical settings and for short durations. Additionally, patients who received intranasal diamorphine or rectal morphine were excluded due to the variable and less predictable bioavailability of these routes, which can result in inconsistent systemic opioid exposure.

The index date was defined as the date of the first opioid administration during hospitalisation. Follow-up time per admission episode was censored at first event date, hospital discharge from the first eligible admission, death or end of study period (24/10/17), whichever came earlier. Eligible admissions required at least one recorded NEWS assessment during the hospital stay. To avoid including the same individual multiple times in the analysis, only the first eligible hospital admission per patient was included in the cohort.

### Data preparation for opioid administration (exposure)

Drug exposure was defined from e-prescribing records that capture if a medication was both prescribed and subsequently administered to the patient by a nurse, as well as drug name, administration route, dosage and timing. Administered opioid exposure was time-varying, with on-drug periods categorised into discrete monotherapy drugs or combination therapy (e.g., oxycodone and morphine co-administered or any other opioid combination). Monotherapy drugs included codeine, tramadol, morphine, fentanyl, buprenorphine patches, oxycodone, and less commonly prescribed opioids were classed as ‘other’, including alfentanil, diamorphine, dihydrocodeine, hydromorphone, meptazinol, and pethidine. We examined if the association with respiratory depression varied between types of opioid drugs administered in hospital. The primary analysis attributed respiratory depression to opioids during a risk window of ‘on drug + 1 day’, unless patients switched to another opioid immediately when the event was attributed to the next opioid drug. For each prescription, daily morphine milligram equivalent (MME/day) was calculated and multiplied by the equivalent analgesic ratio as per the US Centre for Disease Control and Prevention.^17^

Total opioid exposure was categorised into clinically meaningful thresholds [3] as follows: low (< 50 MME/day), moderate (50 to 119 MME/day), high (≥ 120 MME/day) as defined previously [3, 25], and additionally in 30 MME/day increments to explore effects within lower-dose categories. To assess for a flexible, non-linear relationship between MME/day and log-hazard of the outcome, MME/day was also modelled as a continuous variable in a Cox hazards model using restricted cubic splines. Knots were placed at the clinically relevant points mentioned before to capture potential non-linear dose-risk associations.

Crude incidence rates were calculated according to opioid use status, opioid drug, and MME/day.

### Outcome

In the absence of a harmonised, internationally agreed definition of respiratory depression, we used electronically recorded National Early Warning Scores (vital signs) to identify potential events.

Previous work has suggested that a respiratory rate of 12 breaths per minute or lower in a patient who is not physiologically asleep is highly indicative of acute opioid intoxication [26, 27]. Respiratory depression (primary definition) was therefore defined as the first occurrence following index date of any one of the following: respiratory rate (RR) < 10 breaths per minute; RR < 12/min combined with oxygen saturation (O2 saturations) < 94%; RR < 12/min with altered consciousness apart from being alert; OR dispensed naloxone use. Altered consciousness was defined as a patient being responsive only to voice or pain, or being unresponsive, based on the AVPU (Alert, Verbal, Pain, Unresponsive) scale.

For sensitivity analyses, we applied a more stringent definition for severe respiratory depression, defined as the first occurrence of any one of the following: RR < 8 breaths per minute; RR < 10 per minute with O2 saturations < 92%; RR < 10 breaths per minute with altered consciousness; or dispensed naloxone use. An additional sensitivity analysis was performed using a narrow definition of respiratory depression as naloxone administration only.

### Covariates

Baseline characteristics, including age, sex, ethnicity, comorbidities and Townsend deprivation score, were measured and recorded on the date of hospital admission. Comorbidities were defined using ICD-10 codes, as defined by conditions in the Charlson Comorbidity Index (CCI)^18^. Sleep apnoea, renal disease and a history of alcohol excess were chosen additionally as an important covariate for respiratory depression. Townsend deprivation score, a composite measure of material deprivation based on UK census data, was calculated using the first few digits of postcodes^19^. A history of alcohol excess was defined as either a documented referral to alcohol rehabilitation services during admission or prescription of chlordiazepoxide (Librium) during admission, a treatment typically used for managing alcohol withdrawal in cases of moderate to severe dependence.

### Statistical analysis

Descriptive statistics were used to characterise baseline characteristics of the cohort. In patients with more than one respiratory depression event during the study period, only the first (index) event was analysed, and follow-up was censored at the time of the first event. Crude rates per 1,000 person days and Cox proportional hazards models were used to examine comparative risk of administered opioids and respiratory depression. Both unadjusted and adjusted models were fitted. Potential confounders, including age, sex, renal disease, sleep apnoea and a history of alcohol excess, were adjusted for (Additional File 1: Figure S1). Other non-opioid medication exposure, that may impact respiratory depression as effect modifiers such as benzodiazepines and gabapentinoids, were defined using e-prescribing data during admission. Effect modifiers, that were deemed to potentially change the magnitude of the association between the exposure and outcome, included co-administration of gabapentinoids, benzodiazepines and concurrent use of both gabapentinoids and benzodiazepines alongside the administration of opioids.

The primary *‘opioid drug model’* compared the risk of respiratory depression between time-varying opioid administration across different opioid drugs, using codeine as the reference category; and a second analysis using morphine as referent. Secondary analyses included an *‘opioid dose model’* to examine the relationship between time-varying daily MME levels and respiratory depression incidence. MME/day was analysed categorically using the clinically meaningful thresholds [3] mentioned above, and additionally in 30 MME/day increments. MME/day was also modelled as a continuous variable in a Cox hazards model using restricted cubic splines, as described above.

To assess potential effect modification by co-prescription, we fitted an *“opioid interaction”* model including interaction terms between opioid treatment and gabapentinoids use, and between opioid treatment and benzodiazepine use. This model examined the association between time-varying opioid administration with respiratory depression, accounting for concurrent exposure to gabapentinoids and to benzodiazepines. Two-way interactions were estimated to explore whether the effect of opioid exposure was modified by co-exposure to these drugs. We estimated the joint effects relative to a fully unexposed reference group and to opioid exposure alone.

Medication reconciliation documents record and verify a patient’s complete and accurate list of medicines prior to hospital to ensure safe and consistent prescribing during admission, typically performed by the hospital pharmacist. However, this information is not available consistently for all patients depending on the time and length of admission to hospital.

Sensitivity analyses were conducted as follows (i) restricting the cohort to inpatient new opioid users identified through medication reconciliation documents confirming no opioid prescriptions in the previous two years prior to admission; (ii) using more stringent definitions of respiratory depression, including definitions based on naloxone administration alone and severe respiratory depression, as described above; (iii) restricting the cohort to patients with chronic obstructive pulmonary disease (COPD) to assess whether the association between opioid type exposure and respiratory depression differed in this subgroup and (iv) restricting the cohort to patients discharged alive to evaluate the potential impact of death as a competing event. All statistical analyses were conducted in STATA/IC 13.1.

### Ethics

The study received ethics approval from the Health Research Authority (Reference IRAS ID: 190543).

## Results

### Study population and baseline characteristics

**Fig. 1:**
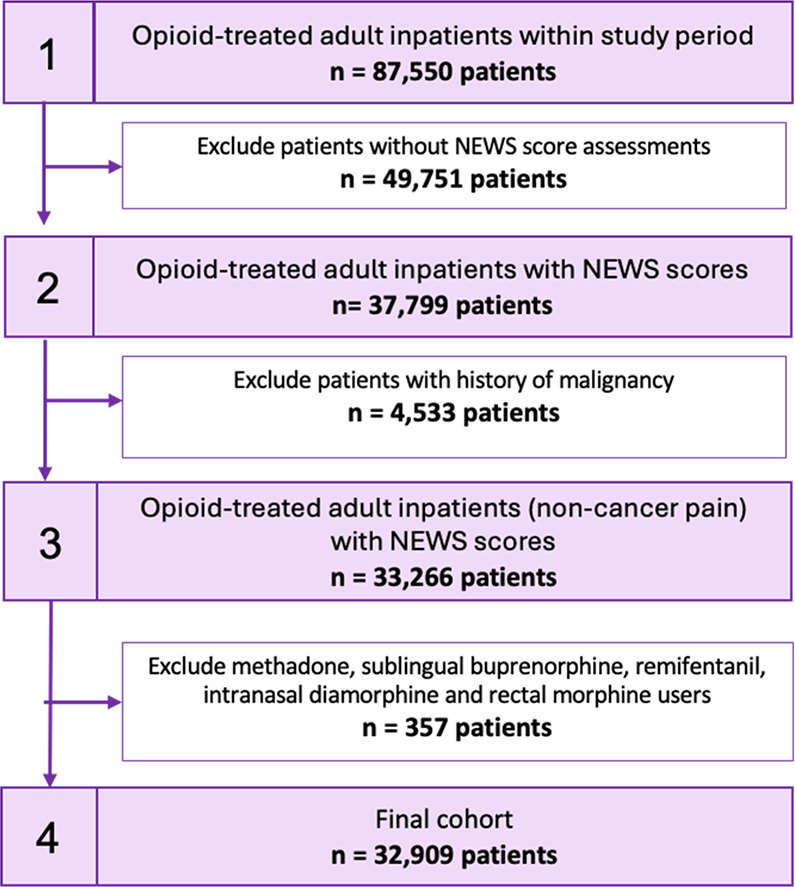
Cohort derivation diagram

Within the study window, a total of 32,909 patients treated with opioids for non-cancer pain met the inclusion criteria and were included in the analysis (mean [SD] age, 61 [20] years) (Fig. 1).

Female patients represented 55% of the cohort, and 92% of patients were of Caucasian ethnicity. A large proportion of patients (44%) were from the most socioeconomically deprived quintile. The most commonly prescribed opioids in hospital at the index date were codeine (35.6%) and morphine (26.1%), followed by combination opioids (19.6%), oxycodone (9.4%), fentanyl (3.8%), tramadol (2.6%), other opioids (2.3%), and buprenorphine patches (0.5%). Codeine, morphine, and combination opioids were more commonly prescribed to younger adults (≤ 55 years), whereas fentanyl, oxycodone, and buprenorphine were predominantly prescribed to older patients (≥ 61 years). Overall, 1,969 opioid users (*6*%) developed respiratory depression (primary definition) during the study period (mean [SD] age, 52.6 [18.3] years). Among the study population, 318 patients (0.9%) received naloxone during their hospital stay (mean [SD] age, 57 [17.5] years). A total of 645 patients (1.9%) experienced severe respiratory depression (stringent definition) (mean [SD] age, 54.6 [17.9] years). Within the cohort 6,227 (18.9%) patients had multiple hospitalisations; for these individuals, only the first hospital admission was included in the analysis. Baseline characteristics are summarised in Table 1.

**Table 1:** Baseline characteristics by opioid drug types

Characteristic		Opioid drug at initiation							
	Total	Codeine	Morphine	Combination	Oxycodone	Fentanyl	Tramadol	Others	Buprenorphine
	*N* = 32,909	11,709 (35.6%)	8,596 (26.1%)	6,464 (19.6%)	3,097 (9.4%)	1,258 (3.8%)	854 (2.6%)	773 (2.3%)	158 (0.5%)
**Age**,** mean [SD] years**	61 (20) years	52 (20) years	51 (19) years	49 (18) years	71 (17) years	61 (20) years	60 (17) years	53 (17) years	78 (13) years
**Age group**									
18–24	2333 (7%)	1059 (9%)	597 (7%)	494 (8%)	41 (1%)	60 (5%)	24 (3%)	58 (8%)	22 (13%) *
25–34	4784 (15%)	1954 (17%)	1371 (16%)	1108 (17%)	103 (3%)	97 (8%)	61 (7%)	88 (11%)	
35–44	4770 (14%)	1662 (14%)	1476 (17%)	1185 (18%)	161 (5%)	114 (9%)	90 (11%)	77 (10%)	
45–54	5600 (17%)	1917 (16%)	1678 (20%)	1274 (20%)	252 (8%)	175 (14%)	140 (16%)	161 (21%)	
55–64	4879 (15%)	1635 (14%)	1486 (17%)	931 (14%)	307 (10%)	176 (14%)	171 (20%)	161 (21%)	
65–74	4413 (13%)	1476 (13%)	914 (11%)	745 (12%)	662 (21%)	269 (21%)	174 (20%)	152 (20%)	21 (13%)
75–84	3764 (11%)	1266 (11%)	645 (8%)	487 (8%)	870 (28%)	237 (19%)	141 (17%)	65 (8%)	53 (34%)
>=85	2366 (7%)	740 (6%)	429 (5%)	240 (4%)	701 (23%)	130 (10%)	53 (6%)	11 (1%)	62 (40%)
**Sex**									
Female	18,186 (55%)	6,367 (54%)	4,655 (54%)	3,645 (56%)	1,877 (61%)	722 (57%)	521 (61%)	291 (38%)	108 (68%)
Male	14,723 (45%)	5,342(46%)	3,941 (46%)	2,819 (44%)	1,220 (40%)	536 (43%)	333 (39%)	482 (62%)	50 (32%)
**Ethnicity**									
Caucasian	30,353 (92%)	10,597 (91%)	7,944 (93%)	6,000 (93%)	2,949 (95%)	1,190 (95%)	807 (95%)	712 (92%)	> 90% *
Non-Caucasian	2,514 (8%)	1,089 (9%)	641 (7%)	458 (7%)	148 (5%)	66 (5%)	47 (5%)	61 (8%)	< 10% *
**Townsend Score**									
1 (Least Deprived)	1186 (4%)	330 (3%)	316 (4%)	273 (4%)	134 (4%)	65 (5%)	31 (4%)	34 (4%)	42 (27%) *
2	3897 (12%)	1186 (10%)	1008 (12%)	748 (12%)	502 (16%)	204 (16%)	117 (14%)	93 (12%)	
3	3580 (11%)	1223 (10%)	942 (11%)	765 (12%)	344 (11%)	135 (11%)	78 (9%)	82 (11%)	11 (7%)
4	9840 (30%)	3507 (30%)	2621 (31%)	1942 (30%)	897 (29%)	368 (29%)	232 (27%)	232 (30%)	41 (26%)
5	14,317 (44%)	5431 (47%)	3682 (43%)	2719 (42%)	1218 (40%)	483 (38%)	394 (46%)	327 (43%)	63 (40%)
***Comorbidities***									
** Myocardial Infarction**	3,941 (12%)	1,209 (10%)	944 (11%)	552 (9%)	708 (23%)	186 (15%)	166 (19%)	113 (15%)	63 (40%)
** Congestive Heart Failure**	2,655 (8%)	804 (7%)	630 (7%)	298 (5%)	558 (18%)	114 (9%)	107 (13%)	93 (12%)	51 (32%)
** Peripheral Vascular Disease**	1,392 (4%)	417 (4%)	299(3%)	195 (3%)	295 (10%)	67 (5%)	59 (7%)	44 (6%)	16 (10%)
** Cerebrovascular Disease**	3,843 (12%)	1,390 (12%)	862 (10%)	467 (7%)	550 (18%)	135 (11%)	152 (18%)	232 (30%)	55 (35%)
** Dementia**	1,542 (5%)	467 (4%)	328 (4%)	137 (2%)	413 (13%)	101 (8%)	36 (4%)	11 (1%)	49 (31%)
** Chronic Pulmonary Disease**	6,737 (21%)	2,193 (12%)	1,645 (19%)	1,261 (20%)	856 (28%)	279 (22%)	274 (32%)	170 (22%)	59 (37%)
** Rheumatologic Disease**	1,102 (3%)	337 (4%)	249 (3%)	194 (3%)	184 (6%)	56 (4%)	53 (6%)	18 (2%)	11 (7%)
** Peptic Ulcer Disease**	700 (2%)	233 (2%)	174 (2%)	124 (2%)	95(3%)	24 (2%)	28 (3%)	22 *	
** Mild Liver Disease**	1,529 (5%)	503 (4%)	384 (4%)	302 (5%)	145 (5%)	47 (4%)	72 (8%)	76 *	
** Diabetes w/o Complications**	3,982 (12%)	1,334 (11%)	838 (10%)	668 (10%)	626 (20%)	186 (15%)	165 (19%)	121 (16%)	44 (28%)
** Diabetes with Complications**	759 (2%)	245 (2%)	141 (2%)	99 (2%)	172 (6%)	36 (3%)	39 (5%)	27 *	
** Hemiplegia or Paraplegia**	1,279 (4%)	373 (3%)	324 (4%)	219 (3%)	143 (5%)	50 (4%)	46 (5%)	124 *	
** Renal Disease**	2,680 (8%)	797 (7%)	436 (5%)	331 (5%)	719 (23%)	179 (14%)	109 (13%)	67 (9%)	42 (27%)
** Severe Liver Disease**	426 (1%)	165 (1%)	91 (1%)	43 (1%)	59 (2%)	18 (1%)	16 (2%)	34 *	
** Obstructive Sleep Apnoea**	730 (2%)	188 (2%)	205 (2%)	143 (2%)	82 (3%)	48 (4%)	35 (4%)	29 *	
** Alcohol Excess**	1,636 (5%)	632 (5%)	358 (4%)	291 (5%)	119 (4%)	40 (3%)	49 (6%)	147 *	
**Morphine Milligram Equivalent (MME)/day on index date**									
< 50	28,787 (87%)	11,708 (99.9%)	7,255 (84%)	4,964 (77%)	2,936 (95%)	683 (54%)	853 (99.9%)	231 (30%)	157 (99.37%)
50 to 119	1,647 (5%)	1 (0.01%)	478 (6%)	929 (14%)	130 (4%)	30 (2%)	1 (0.1%)	77 (9%)	1 (0.6%)
>=120	2,475 (8%)	-	863 (10%)	571 (9%)	31 (1%)	545 (43%)	-	465 (60%)	-

* Records have been grouped or replaced with “NA” for statistical disclosure control to protect patient confidentiality. NA, not available.

### Incidence rates

The incidence rate of respiratory depression was 10.20 (95% CI 9.72 to 10.69) per 1,000 person-days during opioid exposure, compared with 2.41 (95% CI 2.15 to 2.71) per 1,000 person-days in those not on an opioid. Combination opioidshad the highest incidence rate (20.01 [95% CI 18.43 to 21.72]), followed by fentanyl (15.56 [95% CI 12.93 to 18.71]) per 1,000 person-days (Table 2).

### Comparative risk of respiratory depression associated with different opioids

Compared with codeine, after adjustment for confounders, fentanyl (hazard ratio [HR] 3.36, 95% CI 2.70 to 4.18), combination opioids (HR 2.74, 95% CI 2.38 to 3.15), oxycodone (HR 2.10, 95% CI 1.74 to 2.54), and morphine (HR 1.84, 95% CI 1.59 to 2.12) were each associated with a significantly higher risk of respiratory depression. Buprenorphine (topical) was not significantly associated with risk (HR 1.74, 95% CI 0.92 to 3.30), with wide confidence intervals reflecting the small number of events. Similarly, tramadol was not associated with a significantly different risk compared with codeine (HR 0.87, 95% CI 0.55 to 1.37). Full results are presented in Table 2.

Compared with morphine, fentanyl (HR: 1.85, 95% CI 1.50 to 2.27) and combination opioids (HR 1.49, 95% CI 1.32 to 1.69) were associated with a significantly higher risk of respiratory depression after adjustment for confounders. Oxycodone was not associated with a significantly different risk (HR 1.15, 95% CI 0.96 to 1.37). In contrast, codeine (HR 0.54, 95% CI 0.47 to 0.62), tramadol (HR 0.47, 95% CI 0.30 to 0.73), and other opioids (HR 0.20, 95% CI 0.10 to 0.39) were associated with a significantly lower risk compared with morphine (Additional File 2: Table S1).

**Table 2:** Association between administered opioid exposure and respiratory depression

Exposure group	Number of respiratory depression events	Person-days of follow-up time	Incidence rate per 1,000 person-days (95% CI)	Hazard Ratio Unadjusted (95% CI)	Hazard Ratio Adjusted (95% CI) Age + Gender	Hazard Ratio Adjusted (95% CI) Fully Adjusted*	*p*-value	Forest Plot
Noopioid exposure	283	117,259	2.41 (2.15 to 2.71)	1 (Reference)	1 (Reference)	1 (Reference)	-	
Opioid exposure (any)	1,686	165,339	10.20 (9.72 to 10.69)	2.46 (2.17 to 2.79)	2.38 (2.09 to 2.70)	2.32 (2.04 to 2.64)	< 0.001	
**Among patients receiving opioids (reference = codeine)**								
Codeine [Reference]	303	48,157	6.29 (5.62 to 7.04)	1 (Reference)	1 (Reference)	1 (Reference)	-	
Fentanyl	112	7,200	15.56 (12.93 to 18.71)	3.25 (2.61 to 4.04)	3.44 (2.77 to 4.28)	3.36 (2.70 to 4.18)	< 0.001	
Combination	568	28,394	20.01 (18.43 to 21.72)	2.86 (2.49 to 3.29)	2.86 (2.49 to 3.29)	2.74 (2.38 to 3.15)	< 0.001	
Oxycodone	191	30,808	6.20 (5.38 to 7.14)	1.86 (1.55 to 2.24)	2.10 (1.74 to 2.53)	2.10 (1.74 to 2.54)	< 0.001	
Morphine	472	35,350	13.35 (12.20 to 14.62)	1.93 (1.67 to 2.23)	1.90 (1.65 to 2.20)	1.84 (1.59 to 2.12)	< 0.001	
Buprenorphine (Topical)	11	5,298	2.08 (1.15 to 3.75)	1.60 (0.87 to 2.93)	1.90 (1.03 to 3.50)	1.74 (0.92 to 3.30)	0.090	
Tramadol	20	5,464	3.66 (2.36 to 5.67)	0.84 (0.54 to 1.33)	0.89 (0.57 to 1.40)	0.87 (0.55 to 1.37)	0.545	
Others	9	4,629	1.94 (1.01 to 3.73)	0.40 (0.21 to 0.78)	0.38 (0.20 to 0.74)	0.39 (0.20 to 0.76)	0.006	

* Fully adjusted model included adjustment for potential confounders, including age, sex, renal disease, sleep apnoea, and alcohol excess.

### MME/day dosage

In the adjusted MME analysis, patients receiving < 50 MME/day had an incidence rate of 6.4 events (95% CI 6.15 to 6.79) per 1,000 person-days, compared with 14.6 (95% CI 12.96 to 16.43) events per 1,000 person-days among those receiving ≥ 120 MME/day. An MME dose of ≥ 120 was associated with a significantly increased risk of respiratory depression compared with < 50 MME/day (HR 2.06, 95% CI 1.81 to 2.35; Additional File 2: Table S2).

**Fig. 2:**
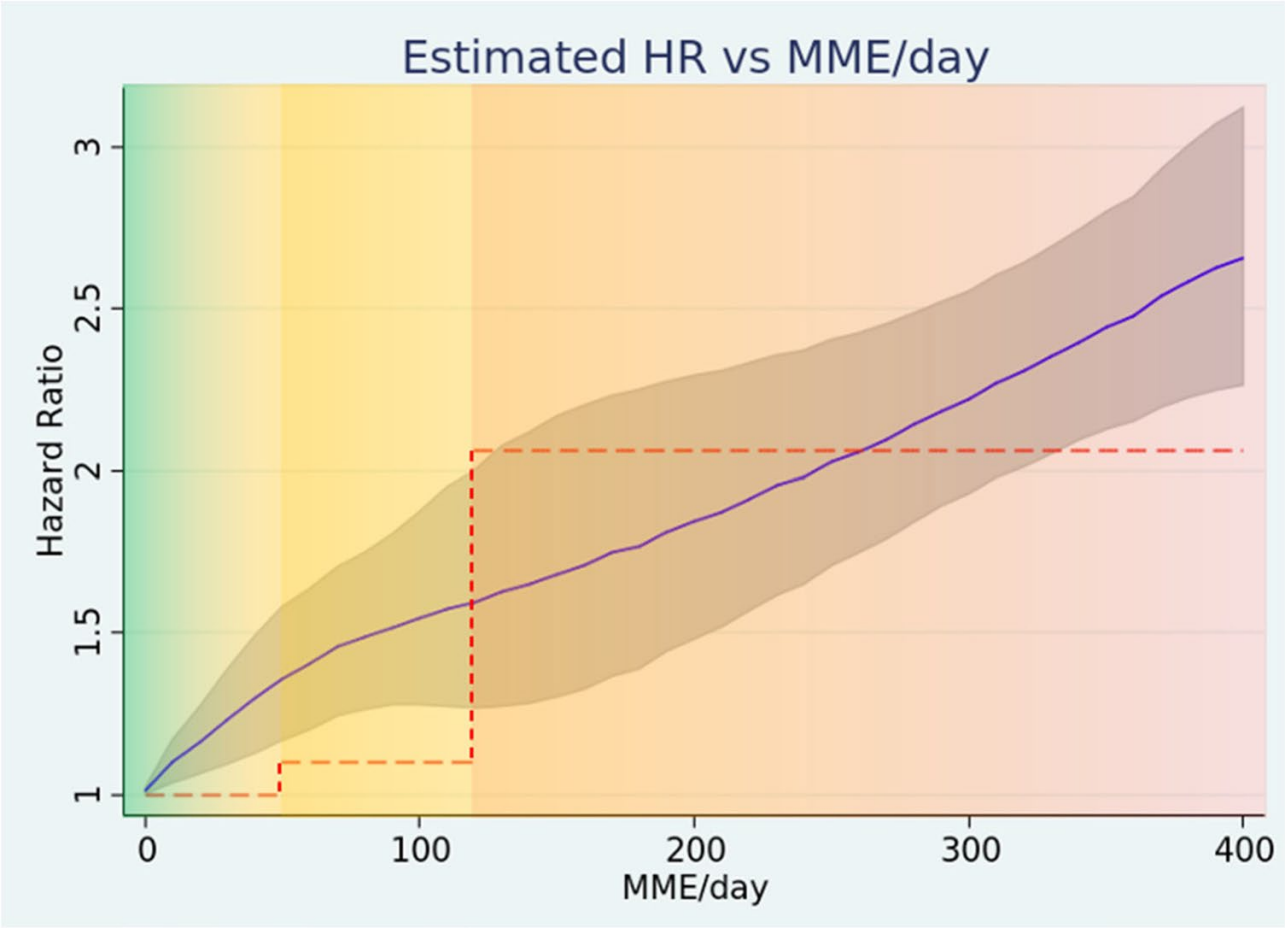
Estimated hazard ratios for respiratory depression by daily opioid dose (MME/day). The blue line represents hazard ratios from a Cox proportional hazards model incorporating restricted cubic splines, with the lowest dose as the reference. Knots were placed at clinically relevant thresholds (0,50,120 and 200 MME/day) to model the non-linear relationship between opioid dose and risk. The red dashed line represents hazard ratios using MME/day as a categorical variable, using 0–50 MME/day as the reference group. While both approaches show increasing risk with higher opioid doses, the spline model provides a more granular representation of the continuous relationship, whereas the categorical analysis simplifies the association within the predefined thresholds

When categorising daily opioid exposure into 30 MME/day increments with < 31 MME/day as the reference, a statistically significant increase in risk was observed at 31 to 60 MME/day (HR 1.18, 95% CI 1.02 to 1.39). Risk generally increased with higher doses, with a more pronounced elevation at 121 to 150 MME/day (HR 1.79, 95% CI 1.06 to 3.02) and 151 to 180 MME/day (HR 2.58, 95% CI 1.60 to 4.16). At higher dose categories, the risk remained elevated, including 241 to 270 MME/day (HR 2.32, 95% CI 1.44 to 3.75) and 301 to 330 MME/day (HR 3.69, 95% CI 2.57 to 5.30) (Additional File 2: Table S3). In the restricted cubic spline analysis, the hazard ratio for respiratory depression shows a non-linear dose-response pattern, demonstrating a progressive increase in risk with increasing opioid dose (Fig. 2). The categorical analysis (red dashed line in Fig. 2), using the 0 to 50 MME/day category as reference, shows similar directionality but oversimplifies and sometimes underestimates the risk within each of the categories (Fig. 2).

### Effect of gabapentinoids and benzodiazepines

The incidence rate of respiratory depression was 9.31 (95% CI 8.36 to 10.37) per 1,000 person-days among patients receiving opioids and gabapentinoids. Concomitant use of opioids and gabapentinoids was associated with a significantly increased risk of respiratory depression compared with opioid use alone after adjustment (HR 1.73, 95% CI 1.53 to 1.96) (Table 3). Compared with unexposed patients, co-exposure to opioids and gabapentinoids was associated with an almost fourfold increase in the risk of respiratory depression (HR 3.88, 95% CI 3.28 to 4.58; Additional File 2: Table S4). Co-prescription of benzodiazepines (without gabapentinoids) was associated with an incidence rate of 5.85 (95% CI 4.48 to 7.64) per 1,000 person-days and associated with lower observed risk (HR 0.65, 95% CI 0.50 to 0.86; Table 3).

**Table 3:** Association between administered opioid exposure, interactions with benzodiazepines, gabapentinoids and respiratory depression

Exposure group	Number of respiratory depression events	Person-days of follow-up	Incidence rate per 1,000 person-days (95% CI)	Hazard Ratio Unadjusted (95% CI)	Hazard Ratio Adjusted (95% CI)	*p*-value	Forest Plot
Interactions of Gabapentinoids with Opioids							
Opioid exposure only	1,355	129,813	10.4 (9.90 to 11.01)	1 (Reference)	-	-	
Opioid + gabapentinoid exposure (without benzodiazepines)	331	35,543	9.31 (8.36 to 10.37)	1.77 (1.6 to 2.0)	1.73 (1.53 to 1.96)	< 0.001	
***Interactions of Benzodiazepines with Opioids***							
Opioid exposure only	1,632	156,095	10.45 (9.96 to 10.97)	1 (Reference)	-	-	
Opioids + benzodiazepine exposure (without gabapentinoids)	54	9,232	5.85 (4.48 to 7.64)	0.59 (0.45 to 0.77)	0.65 (0.50 to 0.86)	0.002	

### Sensitivity analyses

In the new opioid user analysis, 26,085 patients had medicine reconciliation document (79%). 14,238 out of 32,909 patients in the cohort (43%) were identified as new opioid based on medication reconciliation records. Among these, 899 (2.7%) patients experienced the outcome of respiratory depression. Fentanyl was associated with the highest risk, with an adjusted HR of 5.44 (95% CI 3.89 to 7.61), followed by combination opioids (HR 3.80, 95% CI 3.01 to 4.80). Other opioids, including oxycodone (HR 2.82, 95% CI 2.12 to 3.76) and morphine (HR 2.47, 95% CI 1.94 to 3.14), were also associated with significantly increased risks compared with codeine. In contrast, buprenorphine (topical) (HR 2.39, 95% CI 0.95 to 5.98) and tramadol (HR 1.82, 95% CI 0.67 to 4.94) were not associated with a statistically significant increase in risk (Additional File 2: Table S5). For the second sensitivity analysis, using a more stringent definition of respiratory depression (severe respiratory depression or naloxone administration), 645 patients (1.9%) experienced the outcome. After adjustment for confounders, combination opioid use (HR 3.36, 95% CI 2.61 to 4.33), fentanyl (HR 2.61, 95% CI 1.69 to 4.03), oxycodone (HR 2.17, 95% CI 1.57 to 3.00), and morphine (HR 1.72, 95% CI 1.30 to 2.25) were associated with a significantly increased risk compared with codeine. In contrast, buprenorphine (topical) (HR 1.78, 95% CI 0.71 to 4.47), tramadol (HR 0.80, 95% CI 0.35 to 1.83), and other opioids (HR 0.98, 95% CI 0.45 to 2.11) were not associated with a statistically significant difference in risk compared with codeine (Additional File 2: Table S6). When respiratory depression was defined solely as naloxone administration, 318 patients (0.9%) experienced the event. After adjustment for confounders, combination opioid use (HR 4.02, 95% CI 2.75 to 5.87), oxycodone (HR 2.74, 95% CI 1.75 to 4.31), fentanyl (HR 2.78, 95% CI 1.48 to 5.24), and morphine (HR 1.81, 95% CI 1.19 to 2.75) were associated with significantly increased risks compared with codeine. In contrast, buprenorphine (topical) (HR 2.08, 95% CI 0.63 to 6.91), tramadol (HR 0.57, 95% CI 0.14 to 2.37), and other opioids (HR 1.64, 95% CI 0.65 to 4.19) were not associated with a statistically significant difference in risk (Additional File 2: Table S7).

In the sensitivity analysis restricted to patients with COPD, fentanyl was associated with the highest risk of respiratory depression compared with codeine (HR 4.03, 95% CI 2.35 to 6.91), followed by combination opioid use (HR 3.02, 95% CI 2.05 to 4.46) and oxycodone (HR 2.21, 95% CI 1.38 to 3.53). Morphine was also associated with a statistically significant increase in risk (HR 1.58, 95% CI 1.03 to 2.40) compared to codeine. In contrast, buprenorphine (topical) (HR 1.57, 95% CI 0.37 to 6.62), tramadol (HR 0.87, 95% CI 0.54 to 1.39), and other opioids (HR 1.92, 95% CI 0.81 to 4.57) were not associated with statistically significant differences in risk (Additional File 2: Table S8).

Lastly, we conducted a sensitivity analysis restricting the cohort to patients discharged alive and re-estimated the hazard ratios. In our cohort, 916 patients (2.7%) died during admission. In this analysis, fentanyl was associated with the highest risk (HR 3.41, 95% CI 2.74 to 4.25), followed by combination opioids (HR 2.73, 95% CI 2.38 to 3.15), oxycodone (HR 2.08, 95% CI 1.72 to 2.52), and morphine (HR 1.84, 95% CI 1.59 to 2.13), compared with codeine. Buprenorphine (topical) (HR 1.55, 95% CI 0.76 to 3.14) and tramadol (HR 0.88, 95% CI 0.56 to 1.39) were not associated with significant differences in risk compared to codeine (Additional File 2: Table S9).

## Discussion

In this large cohort of hospitalised patients receiving opioids for non-cancer pain, we found that fentanyl, oxycodone, morphine, and combination opioid therapy were associated with a significantly higher risk of respiratory depression compared to codeine after adjustment for confounders, while buprenorphine (topical) was not associated with a statistically significant increase in risk. The effect of fentanyl was more pronounced in the new opioid user sensitivity analysis, being associated with the highest risk (adjusted HR of 5.44 [95% CI: 3.89 to 7.61]). A clear dose-response effect with opioid use was also observed. Using clinically relevant thresholds, doses ≥ 120 MME/day were associated with more than double the risk of respiratory depression compared with < 50 MME/day (HR 2.06, 95% CI 1.81 to 2.35). However, when using finer dose categories, even lower doses (31 to 60 MME/day) were associated with a statistically significant increase in risk compared with < 31 MME/day (HR 1.18, 95% CI 1.02 to 1.39). Compared with opioid use alone, concomitant use of opioids and gabapentinoids was associated with an increased risk of respiratory depression (HR: 1.73, 95% CI 1.53 to 1.96). When compared to no opioid use, concomitant use of opioids and gabapentinoids was associated with an almost fourfold increase in risk (HR: 3.88, 95% CI 3.28 to 4.58).

In our primary analysis, fentanyl was associated with the highest risk of respiratory depression and remained consistently significant across sensitivity analyses. In the new opioid user analysis particularly, the risk of fentanyl was higher, reflecting the higher vulnerability of patients during the initial period of opioid therapy and the potent respiratory depressant effect of this drug. Evidence from related outcomes support our findings, although comparable human studies directly evaluating respiratory depression across non-cancer pain populations are limited. A study evaluating the comparative risk of mortality between opioids in the UK, USA and Canada reported a significantly higher risk among patients prescribed morphine, fentanyl, buprenorphine, oxycodone and combination opioids compared to codeine [28] after confounder adjustment, consistent with our results. Respiratory depression may be the reversible event that precedes opioid-associated deaths highlighting the importance of these findings. Fentanyl, estimated to be 80 to 100 times more potent than morphine [9, 29], binds to µ-opioid receptors which mediate both the analgesic and respiratory depressant effects of opioids [9]. Its high potency, rapid onset, shorter, and greater lipophilicity enable faster brain uptake than morphine or heroin, making it effective for breakthrough pain but also causing abrupt respiration suppression and increased overdose risk. Studies have also shown that fentanyl-related respiratory depression [30] carries a higher risk than heroin and morphine [31], and can reduce cerebral blood flow [32], underscoring the need for clinicians to understand its risks and avoid fentanyl as a first-line potent opioid [33]. The differential increased risks of fentanyl use on respiratory depression are especially important, given the growing problem of increasing use of the drug in several countries including Latin America [34], North America [35, 36] and parts of Europe.

Oxycodone has been a major contributor to opioid-related deaths and a key driver of the opioid epidemic in North America. Smaller one to one comparative studies indicate that oxycodone depresses ventilatory control in a concentration-dependent manner [37], and may be more potent in suppressing breathing compared to tramadol [38], morphine [39], or buprenorphine [40], raising concerns given its widespread prescription. In the UK, oxycodone prescriptions for new users with non-cancer pain increased 30-fold over 12 years in primary care, although not commonly prescribed first line [3]. It is however frequently administered first-line for postsurgical pain in UK hospitals [41], in line with post-surgical guidelines [42]. In contrast, the U.S. prescribes oxycodone more commonly as a first-line opioid compared to other countries for non-cancer pain [1]. Recent pharmacokinetic-pharmacodynamic modelling suggests that oxycodone’s analgesic and respiratory depression effects increase in similar concentrations, unlike fentanyl, which has disproportionately strong respiratory depressant effects [43]. Previous research from Canada indicates that one-third of opioid-related deaths occurred in individuals actively receiving prescription opioids, with oxycodone being the most common [44].

Compared to opioid use alone, concomitant use of gabapentinoids and opioids was associated with an increased risk (HR: 1.73, 95% CI 1.53 to 1.96). A substantial (two-fold) increased mortality risk with the combination of opioids and gabapentinoids has been reported in previous studies in North America [13, 28, 45]. These findings are especially important as in the UK, gabapentinoids frequently continue to be co-prescribed with opioids [46], as prescribers may attempt to use such drug as an “opioid sparing” approach [47]. We observed an apparent lower risk among patients co-prescribed opioids and benzodiazepines (HR: 0.65, 95% CI 0.50 to 0.86). To contextualise this finding, our study period followed years of growing awareness of benzodiazepine-opioid interactions [48] but preceded formal regulatory warnings regarding opioid-gabapentinoid respiratory depression. By this time, benzodiazepines were already recognised as being associated with a higher mortality risk when co-prescribed with opioids [12]. In 2010, benzodiazepines contributed to approximately 30% of all U.S. pharmaceutical related overdose deaths, with co-prescription of opioids involved in the majority (77%) of deaths associated with benzodiazepines [49]. Clinical guidelines had long cautioned against co-prescribing opioids and benzodiazepines [48, 50–52], well before the FDA boxed warning in 2016 [53], and thus, the apparent association observed within this study period may reflect channelling bias due to this awareness. In contrast, gabapentin and pregabalin were commonly perceived as safer alternatives to opioids or benzodiazepines and were often prescribed excessively for various types of pain [54–56], including in high-risk populations. In 2016, gabapentin was the 10th most commonly prescribed medication in the United States [56].

We also observed a dose-response effect with opioids, with risk increasing at higher doses. Doses ≥ 120 MME/day were associated with more than double the risk compared with < 50 MME/day, while even lower doses (31 to 60 MME/day) were associated with a statistically significant increase in risk when compared to < 31 MME/day. Currently, the Faculty of Pain Medicine treatment recommendations in the United Kingdom have a higher threshold of 120 MME/day as the threshold above which harms outweigh benefits [57]. The British Pain Society also recommends a maximum of 120 MME/day in 24 h for chronic pain patients; however, our findings support lowering it. Previous studies have demonstrated the effect of doses > 50 MME/day on mortality [28]. Although the respiratory depressant effect of opioids is documented as dose-dependent [58], we were unable to directly compare our finding on the impact of dosage with existing literature as none of the earlier reviewed studies accounted for the impact of opioid drugs and daily dose on the risk of respiratory depression. It is not common for studies on opioid-related adverse events to report MME/day [7], as it is challenging to prepare such data with a combination of ‘as-required’, missing prescribing/dispensing data and overlapping regular medications [27].

In the sensitivity analysis restricted to patients with COPD, the overall pattern of associations was consistent with the main analysis, although effect sizes were generally larger. Compared with codeine, fentanyl was associated with a fourfold increase in risk, followed by combination opioids (approximately threefold) and oxycodone (approximately twofold). Opioids are commonly prescribed among older patients with comorbid COPD [59, 60], many of whom experience chronic musculoskeletal pain [61]. In a large UK primary care study, we found that musculoskeletal conditions were the leading clinical indication in patients initiated on opioids (80.8%), with substantial overlap between musculoskeletal and respiratory conditions, suggesting that patients with respiratory conditions such as COPD often receive opioids in the context of complex multimorbidity pain [62]. Although some clinical trials have reported that systemic opioids may be used safely in advanced COPD, current respiratory guidelines recommend cautious use [63]. Our findings support the view that patients with COPD may be at increased risk of opioid-related respiratory depression compared with the general population, particularly when exposed to opioids such as fentanyl, oxycodone and combination opioid therapies.

### Strengths

While previous research has focused on pre-clinical murine models [31] or on a small cohorts [64], to the best of our knowledge this is the first study to assess the comparative risk of respiratory depression across multiple opioids in a large representative hospital population cohort. A major strength of this study is the use of clinical records containing vital signs, which allowed a more accurate definition of respiratory depression than reliance on ICD-10 codes alone, the latter being prone to under-ascertainment especially for this outcome. The availability of vital signs further enabled the assessment of different severities of respiratory depression. We also reduced exposure misclassification by using medication administration rather than prescription or pharmacy dispensation records to capture the exact timing of opioid administration by the patient and relevant co-medications. The dataset and availability also enabled the assessment of covariates such as alcohol excess in a more robust manner than diagnostic codes alone. Additionally, our dataset allowed for time-varying analyses, including concomitant administration of gabapentinoids and benzodiazepines.

### Limitations

Several important limitations need to be acknowledged. First, the sensitivity analysis restricted to new opioid users was limited to the subset of patients with available medication reconciliation data, as the identification of opioid naïve patients relied on medication reconciliation records, which were not consistently available for all admissions. Second, NEWS measurements may have been inconsistently captured during hospitalisations, potentially missing some events. Third, we did not include dose information for gabapentinoids and benzodiazepines when analysing concurrent medications. Given the dose-dependent effect of these drug classes on opioids, this could be relevant in future analyses. Comorbidities that were adjusted for did not include their severity, which is a limitation in general of administrative and EHR data. We were unable to adjust for low BMI or frailty due to these variables being inconsistently measured in the dataset. Lastly, estimates for topical buprenorphine, tramadol and other less frequently prescribed opioids should be interpreted cautiously due to the small number of exposed patients and events, which limited the precision of the estimated associations.

### Wider implications

Despite these limitations, our results carry important clinical implications. Fentanyl was associated with the highest observed risk of respiratory depression, alongside commonly prescribed opioids in hospitals post-surgery such as oxycodone [41], and combination opioid therapy. The increased risk with concomitant gabapentinoids, together with a dose-response relationship with opioids (where risk significantly increased at 31 to 60 MME/day relative to < 31 MME/day, and more than doubled at ≥ 120 MME/day compared with < 50 MME/day, provides important information to support safer prescribing practices.

## Conclusions

In this large cohort study evaluating the comparative risk of respiratory depression across different opioid drugs in patients with non-cancer pain in Northwest England, we utilised hospital EHRs with vital sign measurements to define respiratory depression, alongside drug administration records to more accurately capture opioid exposure. Compared with codeine, the administration of fentanyl, combination opioids, oxycodone and morphine were associated with a significantly higher risk of respiratory depression, while buprenorphine (topical) was not associated with a statistically significant increase in risk. Relative to morphine, fentanyl and combination opioids showed a significantly higher risk. In new opioid users, the respiratory depression risk of fentanyl administration was more pronounced. Analysis of MME/day using restricted cubic splines showed a clear dose-response relationship. Risk increased significantly at 31 to 60 MME/day relative to < 31 MME/day and more than doubled at ≥ 120 MME/day relative to < 50 MME/day. Compared to opioid use alone, concomitant use of gabapentinoids and opioids was associated with an increased risk. These results emphasise the particularly elevated risk of fentanyl relative to other opioids, the impact of polypharmacy, and the dose-dependent effect of opioid-related respiratory depression. Our findings underscore the importance of tailored prescribing and careful monitoring of patients with these high-risk factors for respiratory depression, especially in non-cancer pain populations where alternative treatments (non-opioid analgesics, non-pharmacological approaches) should be prioritised whenever possible.

## Supplementary Information

Below is the link to the electronic supplementary material.

Supplementary Material 1: Additional File 1: Figure S1. Directed Acyclic Graph demonstrating potential effect of confounders and effect modifiers for opioid drug administration analysis.

Supplementary Material 2: Additional File 2: Tables S1–S9.

## Data Availability

The data that support the findings of this study are available from the Northern Care Alliance, but restrictions apply to the availability of these data to protect patient re-identification and are not publicly available. Data used for this study are however available with permission of the Northern Care Alliance (https://www.northerncarealliance.nhs.uk/contact-us). In order to access the data, researchers will need to be Office of National Statistics Safe Researcher accredited and need to have undergone appropriate background checks prior to permission being granted from the host organisation.

## Abbreviations

AVPU: Alert, Verbal, Pain, Unresponsive
BMI: Body Mass Index
CCI: Charlson Comorbidity Index
CI: Confidence Interval
MME/day: Daily morphine milligram equivalent
EHR: Electronic Health Records
FDA: Food and Drug Administration
HR: Hazard Ratio
NEWS: National Early Warning Score
NA: Not Available
RR: Respiratory rate
SD: Standard Deviation
UK: United Kingdom
