# Supplementary material for: Opioid-specific risk of respiratory depression in non-cancer pain: a retrospective cohort study
Source: BMC Med. 2026 Jul 8;24:380. doi: 10.1186/s12916-026-04972-z (PMC13343915; doi:10.1186/s12916-026-04972-z)
Supplement: Supplementary file 1 — Supplementary Material 1: Additional File 1: Figure S1. Directed Acyclic Graph demonstrating potential effect of confounders and effect modifiers for opioid drug administration analysis. [file 12916_2026_4972_MOESM1_ESM.docx]

**Additional File 1**

**Figure S1: Directed Acyclic Graph demonstrating potential effect of confounders and effect modifiers for opioid drug administration analysis**


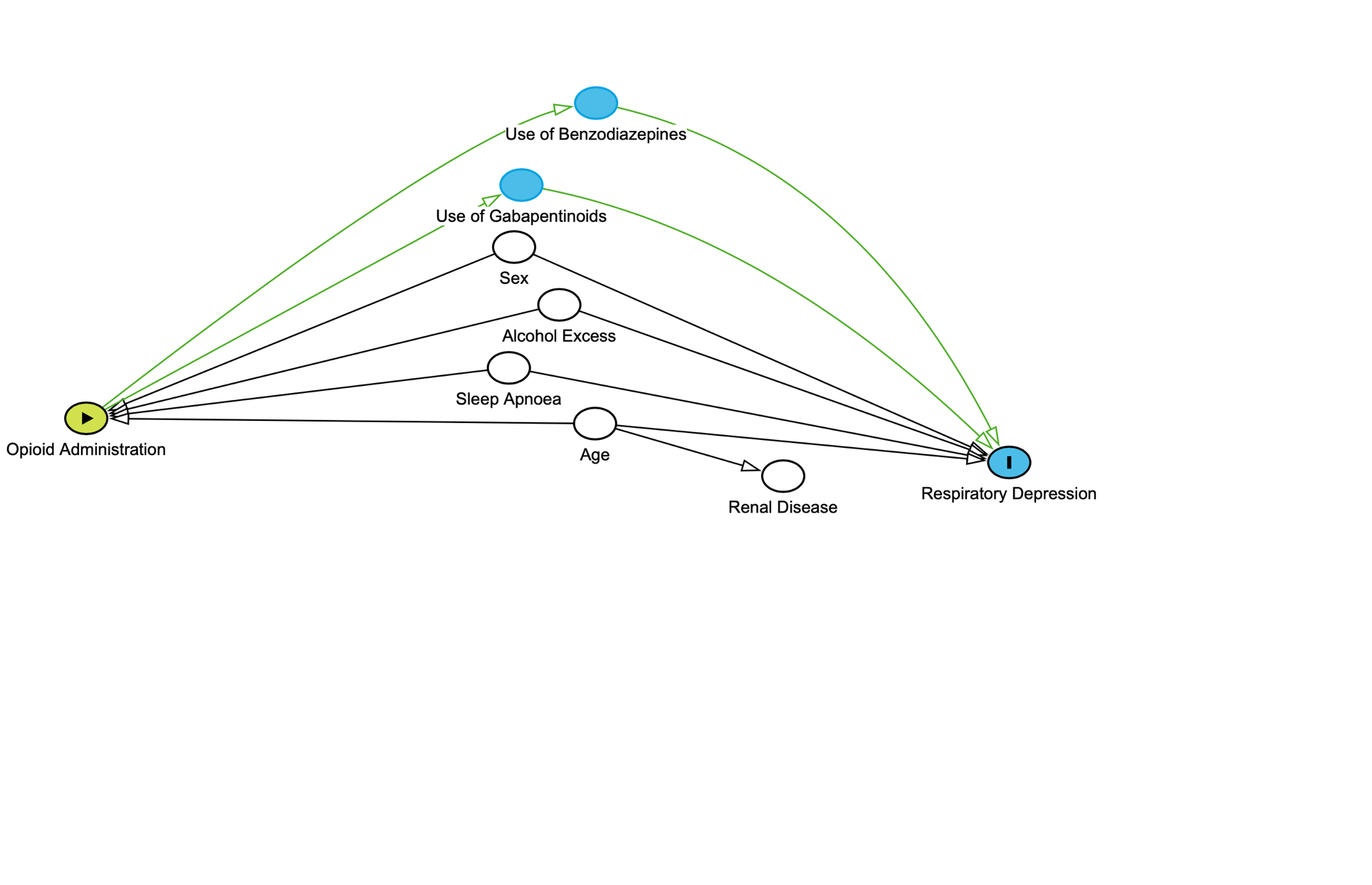
**
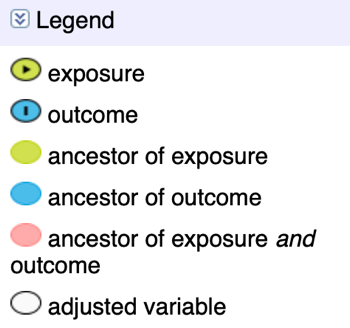
**

**
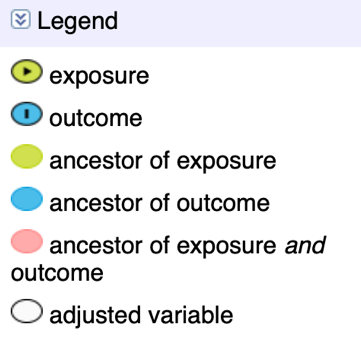
**

Potential confounders, including renal disease and sleep apnoea, were adjusted for in the analysis, as they were associated with both opioid **drug** administration (exposure) and respiratory depression (outcome), but not on the causal pathway. Effect modifiers were classed as gabapentinoids and benzodiazepines, that could change the magnitude of the association between the exposure and outcome.
