# Supplementary material for: Opioid-specific risk of respiratory depression in non-cancer pain: a retrospective cohort study
Source: BMC Med. 2026 Jul 8;24:380. doi: 10.1186/s12916-026-04972-z (PMC13343915; doi:10.1186/s12916-026-04972-z)
Supplement: Supplementary file 2 — Supplementary Material 2: Additional File 2: Tables S1–S9. [file 12916_2026_4972_MOESM2_ESM.docx]

**Table S1. Association between administered opioid exposure and respiratory depression (morphine as reference)**

| **Exposure group** | | **Number of respiratory depression events** | **Person-days of follow-up** | **Incidence rate per 1,000 person-days** | **Hazard Ratio Unadjusted (95% CI)** | **Hazard Ratio Adjusted (95% CI)** | **p-value** | **Forest Plot** |
| --- | --- | --- | --- | --- | --- | --- | --- | --- |
| ***Opioid Drugs*** | | | | | | | | 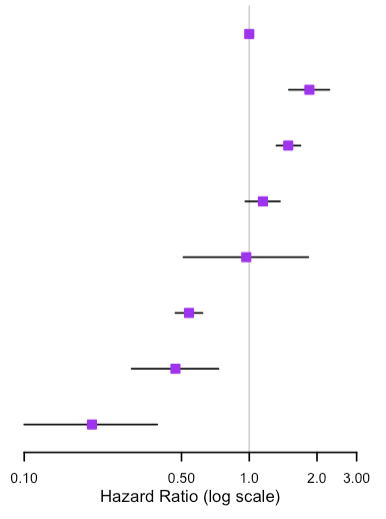 |
| ***Opioid Drugs*** | **Morphine [Reference]** | 472 | 35,350 | 13.35 (12.20–14.62) | 1 (Reference) | 1 (Reference) | - |  |
|  | **Fentanyl** | 112 | 7,200 | 15.56 (12.93–18.71) | 1.68 (1.37 to 2.07) | 1.85 (1.50 to 2.27) | <0.001 |  |
|  | **Combination** | 568 | 28,394 | 20.01 (18.43–21.72) | 1.48 (1.31 to 1.67) | 1.49 (1.32 to 1.69) | <0.001 |  |
|  | **Oxycodone** | 191 | 30,808 | 6.20 (5.38–7.14) | 0.96 (0.81 to 1.14) | 1.15 (0.96 to 1.37) | 0.133 |  |
|  | **Buprenorphine (Topical)** | 11 | 5,298 | 2.08 (1.15–3.75) | 0.83 (0.45 to 1.51) | 0.97 (0.51 to 1.83) | 0.923 |  |
|  | **Codeine** | 303 | 48,157 | 6.29 (5.62–7.04) | 0.52 (0.45 to 0.60) | 0.54 (0.47 to 0.62) | <0.001 |  |
|  | **Tramadol** | 20 | 5,464 | 3.66 (2.36–5.67) | 0.44 (0.28 to 0.68) | 0.47 (0.30 to 0.73) | 0.001 |  |
|  | **Others** | 9 | 4,629 | 1.94 (1.01–3.73) | 0.21 (0.11 to 0.40) | 0.20 (0.10 to 0.39) | <0.001 |  |
|  | | | | | | | |  |

**Table S2. Association between clinically defined Morphine Milligram Equivalent (MME) daily thresholds and respiratory depression**

| **Exposure measure** | **MME/day** | **Number of respiratory depression events** | **Person-days of follow-up time** | **Incidence rate per 1,000 person-days** | **Hazard Ratio Unadjusted**  **(95% CI)** | **Hazard Ratio Adjusted (95% CI)**  **Age + Sex** | **Hazard Ratio Adjusted (95% CI)**  **Fully Adjusted** | **p-value** | **Forest Plot** |
| --- | --- | --- | --- | --- | --- | --- | --- | --- | --- |
| Opioid dose (MME) | <50 | 1,592 | 246,196 | 6.47  (6.15 to 6.79) | 1  (Reference) | 1  (Reference) | 1  (Reference) | — | 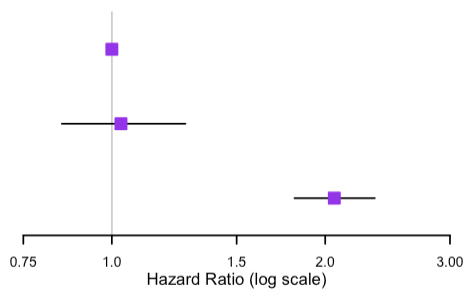 |
|  | 50 to <120 | 104 | 17,629 | 5.90  (4.87 to 7.15) | 1.13  (0.92 to 1.37) | 1.06  (0.87 to 1.29) | 1.03  (0.85 to 1.27) | 0.725 |  |
|  | ≥120 | 273 | 18,711 | 14.59  (12.96 to 16.43) | 2.21  (1.93 to 2.51) | 2.12  (1.86 to 2.41) | 2.06  (1.81 to 2.35) | <0.001 |  |
|  | | | | | | | | |  |

**Table S3. Association between Morphine Milligram Equivalent (MME) daily dose categories (30 mg increments) and respiratory depression**

| **Exposure measure** | **Category** | **Hazard Ratio (95% CI)** | **Forest Plot** |
| --- | --- | --- | --- |
| Opioid dose (MME) | <31 MME/day | 1.00 (Reference) | 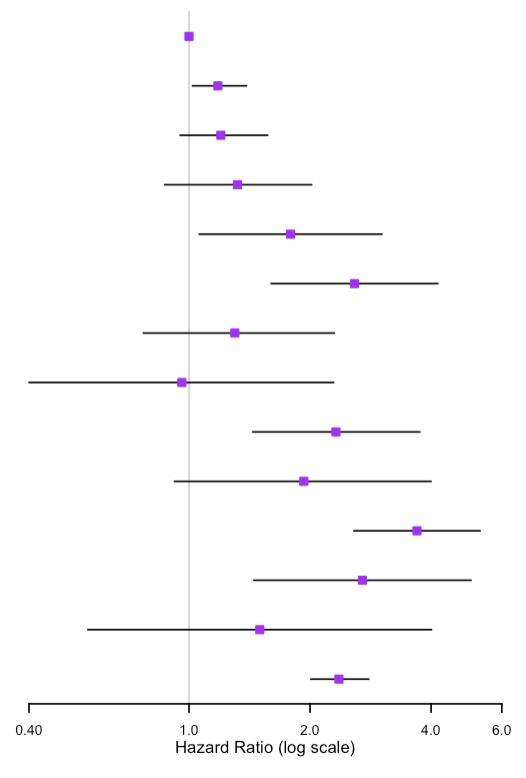 |
|  | 31–60 MME/day | 1.18 (1.02 to 1.39) |  |
|  | 61–90 MME/day | 1.20 (0.95 to 1.57) |  |
|  | 91–120 MME/day | 1.32 (0.87 to 2.02) |  |
|  | 121–150 MME/day | 1.79 (1.06 to 3.02) |  |
|  | 151–180 MME/day | 2.58 (1.60 to 4.16) |  |
|  | 181–210 MME/day | 1.30 (0.77 to 2.30) |  |
|  | 211–240 MME/day | 0.96 (0.40 to 2.29) |  |
|  | 241–270 MME/day | 2.32 (1.44 to 3.75) |  |
|  | 271–300 MME/day | 1.93 (0.92 to 4.00) |  |
|  | 301–330 MME/day | 3.69 (2.57 to 5.30) |  |
|  | 331–360 MME/day | 2.70 (1.45 to 5.03) |  |
|  | 361–390 MME/day | 1.50 (0.56 to 4.01) |  |
|  | 391–420 MME/day | 2.36 (2.01 to 2.80) |  |
|  | | |  |

**Table S4. Association between administered opioid exposure, interactions of gabapentinoids / benzodiazepines with opioids.**

| **Exposure group** | **Hazard Ratio Unadjusted (95% CI)** | **Hazard Ratio Adjusted (95% CI)** | **p-value** | **Forest Plot** |
| --- | --- | --- | --- | --- |
| ***Interactions of Gabapentinoids with Opioids*** | | | | |
| Off drug | 1 (Reference) | - | - | 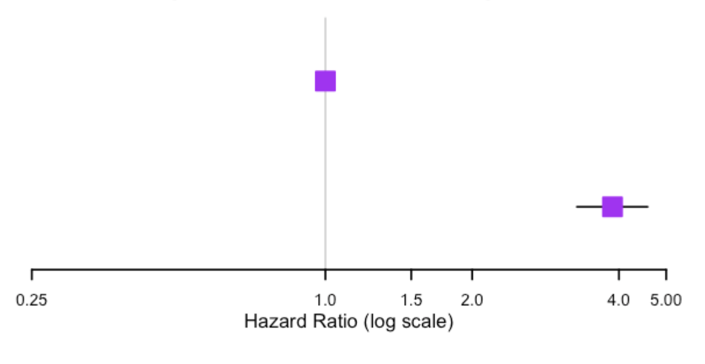 |
| On opioids + Gabapentinoids  (without benzodiazepines) | 4.19 (3.55 to 4.94) | 3.88 (3.28 to 4.58) | <0.001 |  |
|  | | | |  |
| ***Interactions of Benzodiazepines with Opioids*** | | | | |
| Off drug | 1 (Reference) | - | - | ***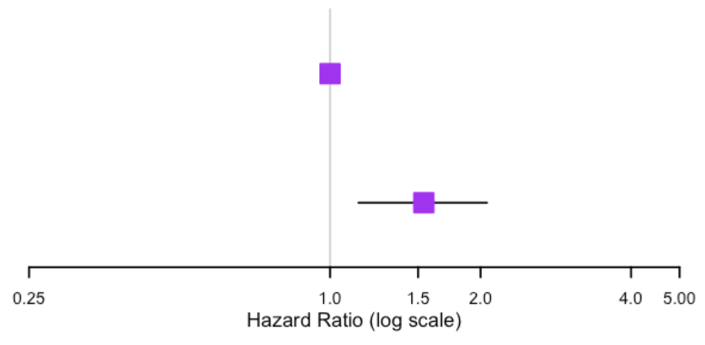*** |
| On opioids + Benzodiazepines  (without gabapentinoids) | 1.47 (1.10 to 1.97) | 1.54 (1.14 to 2.06) | 0.004 |  |
|  | | | |  |

**Table S5. Association between administered opioid exposure and respiratory depression (codeine as reference) for new opioid users only.**

| **Exposure group** | | **Number of respiratory depression events** | **Incidence rate per 1,000 person days** | **Hazard Ratio Unadjusted (95% CI)** | **Hazard Ratio Fully Adjusted (95% CI)** | **p-value** | **Forest Plot** |
| --- | --- | --- | --- | --- | --- | --- | --- |
| **Not opioid exposure** | | 179 | 2.18 | 1 (Reference) | - | - | 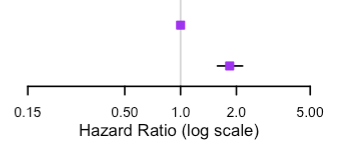 |
| **Opioid exposure (any)** | | 720 | 8.80 | 1.87 (1.61 to 2.18) | 1.84 (1.58 to 2.16) | <0.0001 |  |
| ***Opioid Drugs*** | | | | | | |  |
| ***Opioid Drugs*** | **Codeine [Reference]** | 99 | 3.66 | 1 (Reference) | - | - | 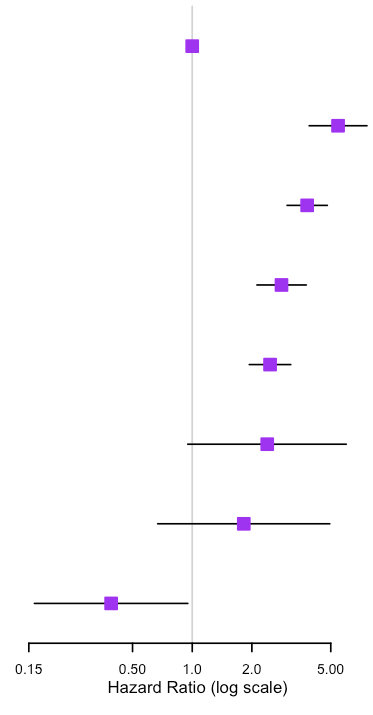 |
|  | **Fentanyl** | 53 | 24.18 | 5.11 (3.66 to 7.13) | 5.44 (3.89 to 7.61) | <0.0001 |  |
|  | **Combination** | 250 | 22.08 | 3.85 (3.06 to 4.87) | 3.80 (3.01 to 4.80) | <0.0001 |  |
|  | **Oxycodone** | 101 | 6.15 | 2.49 (1.89 to 3.29) | 2.82 (2.12 to 3.76) | <0.0001 |  |
|  | **Morphine** | 203 | 11.82 | 2.52 (1.98 to 3.21) | 2.47 (1.94 to 3.14) | <0.0001 |  |
|  | **Buprenorphine (Topical)** | 5 | 1.71 | 2.05 (0.83 to 5.12) | 2.39 (0.95 to 5.98) | 0.063 |  |
|  | **Tramadol** | 4 | 3.65 | 1.69 (0.62 to 4.59) | 1.82 (0.67 to 4.94) | 0.242 |  |
|  | **Others** | 5 | 1.52 | 0.41 (0.17 to 1.02) | 0.39 (0.16 to 0.95) | 0.038 |  |
|  | | | | | | |  |

**Table S6. Association between administered opioid exposure and severe respiratory depression or naloxone administration (codeine as referent)**

| **Exposure group** | | **Number of respiratory depression events** | **Hazard Ratio Unadjusted (95% CI)** | **Hazard Ratio Adjusted (95% CI)** | **p-value** | **Forest Plot** |
| --- | --- | --- | --- | --- | --- | --- |
| **Not opioid exposure** | | 120 | 1 (Reference) | 1 (Reference) | - | 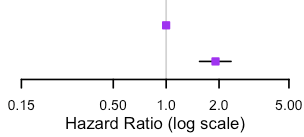 |
| **Opioid exposure (any)** | | 525 | 1.98 (1.62 to 2.43) | 1.91 (1.55 to 2.34) | 0.001 |  |
|  | | | | | |  |
|  | | | | | |  |
| ***Opioid Drugs*** | **Codeine [Referent]** | 87 | 1 (Reference) | 1 (Reference) | - | 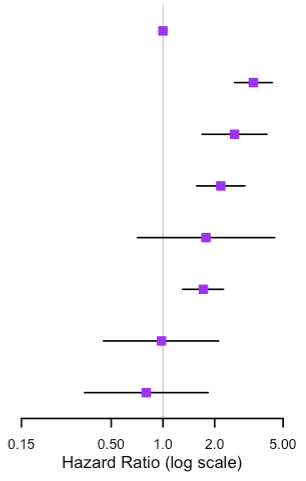 |
|  | **Combination** | 196 | 3.49 (2.71 to 4.50) | 3.36 (2.61 to 4.33) | <0.0001 |  |
|  | **Fentanyl** | 27 | 2.58 (1.67 to 3.97) | 2.61 (1.69 to 4.03) | <0.0001 |  |
|  | **Oxycodone** | 71 | 2.03 (1.48 to 2.78) | 2.17 (1.57 to 3.00) | <0.0001 |  |
|  | **Buprenorphine (Topical)** | 6 | 1.89 (0.82 to 4.40) | 1.78 (0.71 to 4.47) | 0.218 |  |
|  | **Morphine** | 125 | 1.80 (1.37 to 2.36) | 1.72 (1.30 to 2.25) | <0.0001 |  |
|  | **Others** | 7 | 0.99 (0.46 to 2.15) | 0.98 (0.45 to 2.11) | 0.950 |  |
|  | **Tramadol** | 6 | 0.80 (0.35 to 1.83) | 0.80 (0.35 to 1.83) | 0.602 |  |
|  | | | | | |  |

**Table S7. Association between administered opioid exposure and naloxone administration only (codeine as reference)**

| **Exposure group** | | **Number of respiratory depression events** | **Hazard Ratio Unadjusted (95% CI)** | **Hazard Ratio Adjusted (95% CI)** | **p-value** | **Forest Plot** |
| --- | --- | --- | --- | --- | --- | --- |
| **Not opioid exposure** | | 58 | 1 (Reference) | - | - | 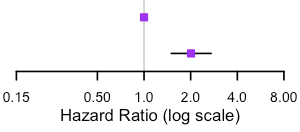 |
| **Opioid exposure (any)** | | 260 | 2.02 (1.51- 2.70) | 2.01 (1.50-2.71) | <0.0001 |  |
|  | | | | | |  |
|  | |  |  |  |  |  |
| ***Opioid Drugs*** | **Codeine [Reference]** | 37 | 1 (Reference) | 1 (Reference) | - | 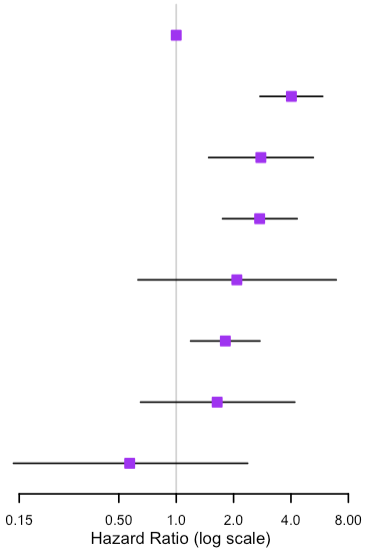 |
|  | **Combination** | 99 | 4.16 (2.85 to 6.06) | 4.02 (2.75 to 5.87) | <0.0001 |  |
|  | **Fentanyl** | 13 | 2.91 (1.54 to 5.47) | 2.78 (1.48 to 5.24) | 0.002 |  |
|  | **Oxycodone** | 45 | 2.85 (1.83 to 4.43) | 2.74 (1.75 to 4.31) | <0.0001 |  |
|  | **Buprenorphine (Topical)** | 4 | 2.83 (0.99 to 8.10) | 2.08 (0.63 to 6.91) | 0.230 |  |
|  | **Morphine** | 55 | 1.86 (1.22 to 2.82) | 1.81 (1.19 to 2.75) | 0.005 |  |
|  | **Others** | 5 | 1.58 (0.62 to 4.03) | 1.64 (0.65 to 4.19) | 0.299 |  |
|  | **Tramadol** | 2 | 0.59 (0.14 to 2.46) | 0.57 (0.14 to 2.37) | 0.440 |  |
|  | | | | | |  |

**Table S8. Association between administered opioid exposure and respiratory depression (codeine as reference) for patients diagnosed with COPD**

| **Exposure group** | **Hazard Ratio Adjusted (95% CI)**  **Fully Adjusted** | **P-value** |
| --- | --- | --- |
| Codeine [Reference] | 1 (Reference) | - |
| Fentanyl | 4.03 (2.35 to 6.91) | <0.0001 |
| Combination | 3.02 (2.05 to 4.46) | <0.0001 |
| Oxycodone | 2.21 (1.38 to 3.53) | <0.0001 |
| Morphine | 1.58 (1.03 to 2.40) | 0.034 |
| Buprenorphine (Topical) | 1.57 (0.37 to 6.62) | 0.541 |
| Tramadol | 0.87 (0.54 to 1.39) | 0.658 |
| Others | 1.92 (0.81 to 4.57) | 0.138 |
| **Exposure measure**  **MME/day** | **Hazard Ratio Adjusted (95% CI)**  **Fully Adjusted** | **P-value** |
| <50 | 1 (Reference) | - |
| 50 to <120 | 0.90 (0.54 to 1.50) | 0.690 |
| ≥120 | 2.25 (1.65 to 3.08) | <0.0001 |
| **Exposure group** | **Hazard Ratio Adjusted (95% CI)**  **Fully Adjusted** | **P-value** |
| On Opioids | 1 (Reference) | - |
| On Opioids and Gabapentinoids | 1.45 (1.06 to 1.99) | 0.020 |
| **Exposure group** | **Hazard Ratio Adjusted (95% CI)**  **Fully Adjusted** | **P-value** |
| On Opioids | 1 (Reference) | - |
| On Opioids and Benzodiazepines | 0.65 (0.33 to 1.27) | 0.207 |

**Table S9. Association between administered opioid exposure and respiratory depression (codeine as referent) restricted to patients discharged alive**

| **Exposure group** | **Hazard Ratio Adjusted (95% CI)**  **Fully Adjusted** | **P-value** |
| --- | --- | --- |
| Not opioid exposure | 1 (Reference) | - |
| Opioid exposure (any) | 2.26 (1.98 to 2.58) | <0.0001 |
|  | | |
| Codeine [Referent] | 1 (Reference) | - |
| Fentanyl | 3.41 (2.74 to 4.25) | <0.0001 |
| Combination | 2.73 (2.38 to 3.15) | <0.0001 |
| Oxycodone | 2.08 (1.72 to 2.52) | <0.0001 |
| Morphine | 1.84 (1.59 to 2.13) | <0.0001 |
| Buprenorphine (Topical) | 1.55 (0.76 to 3.14) | 0.230 |
| Tramadol | 0.88 (0.56 to 1.39) | 0.609 |
| Others | 0.25 (0.10 to 0.59) | 0.002 |
| **Exposure measure**  **MME/day** | **Hazard Ratio Adjusted (95% CI)**  **Fully Adjusted** | **P-value** |
| <50 | 1 (Reference) | - |
| 50 to <120 | 1.04 (0.85 to 1.27) | 0.727 |
| ≥120 | 2.06 (1.81 to 2.35) | <0.0001 |
| **Exposure group** | **Hazard Ratio Adjusted (95% CI)**  **Fully Adjusted** | **P-value** |
| On Opioids | 1 (Reference) | - |
| On Opioids and Gabapentinoids | 1.55 (1.12 to 2.13) | 0.007 |
| **Exposure group** | **Hazard Ratio Adjusted (95% CI)**  **Fully Adjusted** | **P-value** |
| On Opioids | 1 (Reference) | - |
| On Opioids and Benzodiazepines | 0.66 (0.51 to 0.87) | 0.003 |
